# Inhibitory Effects of Urolithins, Bioactive Gut Metabolites from Natural Polyphenols, against Glioblastoma Progression

**DOI:** 10.3390/nu15234854

**Published:** 2023-11-21

**Authors:** Ching-Kai Shen, Bor-Ren Huang, Vichuda Charoensaensuk, Liang-Yo Yang, Cheng-Fang Tsai, Yu-Shu Liu, Sheng-Wei Lai, Dah-Yuu Lu, Wei-Lan Yeh, Chingju Lin

**Affiliations:** 1 Graduate Institute of Biomedical Science, China Medical University, Taichung 404328, Taiwan; 107305208@365.cmu.edu.tw; 2 School of Medicine, Tzu Chi University, Taichung 404, Taiwan; 3 Department of Neurosurgery, Taichung Tzu Chi Hospital, Buddhist Tzu Chi Medical Foundation, Taichung 404, Taiwan; 4 Department of Pharmacology, School of Medicine, China Medical University, Taichung 404328, Taiwan; 5 Department of Physiology, School of Medicine, China Medical University, Taichung 40402, Taiwan; 6 Laboratory for Neural Repair, China Medical University Hospital, Taichung 404327, Taiwan; 7 Department of Medical Laboratory Science and Biotechnology, Asia University, Taichung 41354, Taiwan; tsaicf@asia.edu.tw; 8 Department of Photonics and Communication Engineering, Asia University, Taichung 41354, Taiwan; 9 Department of Biochemistry, School of Medicine, China Medical University, Taichung 40402, Taiwan; wlyeh@mail.cmu.edu.tw; 10 Institute of New Drug Development, China Medical University, Taichung 40402, Taiwan

**Keywords:** urolithins, AhR, glioblastoma, VCAM-1, PD-L1

## Abstract

We previously reported that proinflammatory cytokines, particularly tumor necrosis factor (TNF)-α, promoted tumor migration, invasion, and proliferation, thus worsening the prognosis of glioblastoma (GBM). Urolithins, the potent metabolites produced by the gut from pomegranate polyphenols, have anticancer properties. To develop an effective therapy for GBM, this study aimed to study the effects of urolithins against GBM. Urolithin A and B significantly reduced GBM migration, reduced epithelial–mesenchymal transition, and inhibited tumor growth. Moreover, urolithin A and B inhibited TNF-α-induced vascular cell adhesion molecule (VCAM)-1 and programmed death ligand 1 (PD-L1) expression, thereby reducing human monocyte (HM) binding to GBM cells. Aryl hydrocarbon receptor (AhR) level had higher expression in patients with glioma than in healthy individuals. Urolithins are considered pharmacological antagonists of AhR. We demonstrated that the inhibition of AhR reduced TNF-α-stimulated VCAM-1 and PD-L1 expression. Furthermore, human macrophage condition medium enhanced expression of PD-L1 in human GBM cells. Administration of the AhR antagonist attenuated the enhancement of PD-L1, indicating the AhR modulation in GBM progression. The modulatory effects of urolithins in GBM involve inhibiting the Akt and epidermal growth factor receptor pathways. The present study suggests that urolithins can inhibit GBM progression and provide valuable information for anti-GBM strategy.

## 1. Introduction

GBM is a serious primary malignant central nervous system tumor in adults because of its rapid growth rate and extensive invasion of surrounding tissue [1]. A heterogenous tumor microenvironment (TME) and the adaptive nature of GBMs are the major contributors to treatment ineffectiveness [2]. The TME comprises both cancerous and noncancerous cells that contribute to the progression of GBM [3]. The majority of noncancerous TME constituents are microglia and peripheral monocytes/macrophages (so-called tumor-associated macrophages (TAMs)), which constitute as many as 30–50% of cells in GBM tissue [4]. A study demonstrated a positive correlation between the expression of CD11b-positive myeloid cell populations and tumor proliferation in human glioma, suggesting the regulatory role of TAMs in tumor progression [5]. In addition, tumor-infiltrating myeloid cells contribute to the expression of immune checkpoint programmed death ligand 1 (PD-L1), which negatively regulates T-cell functions in GBM [6]. Patients with GBM have higher levels of PD-L1 in their peripheral blood monocytes and tumor-infiltrating macrophages, which induce autologous T-cell apoptosis [7]. Clinically, the increased PD-L1 expression in patients with GBM correlates with poor prognosis. Patients with GBM express higher levels of PD-L1 than those with grade I, II, or III glioma [8]. Therefore, targeting the suppression of tumor growth and management of monocyte/macrophage infiltration would benefit the development of therapeutic strategies for GBM.

Pomegranate has strong antioxidative and anti-inflammatory effects and can be used to treat various chronic diseases [9,10,11]. Pomegranate contains several phytochemical compounds such as hydrolysable tannins (ellagitannins and gallotannins), condensed tannins, flavonoids, and phenolic acids that confer therapeutic effects [12].Urolithins are metabolites obtained from gut microbiota following the consumption of ellagic acid or ellagitannin obtained from pomegranate [13,14]. Urolithin A and urolithin B are a class of secondary polyphenolic metabolites produced by the gut microbiota–mediated degradation of ellagitannins and ellagic acid-abundant foods such as pomegranates [15]. Urolithin A can repair damaged neurons, suppress the apoptosis of hippocampal cells, and reduce amyloid β levels [16]. Urolithin A has neuroprotective effects against Parkinson’s disease and brain aging [17,18]. Urolithin B reduced inflammatory cytokines in the small intestine of aging mice [19] and in a mouse model of D-gal-induced Alzheimer’s disease [20]. Urolithin A also strongly suppresses the cell proliferation of a variety of cancers [21,22,23,24,25]. Although numerous reports have shown the inhibitory effects of urolithin A against different tumors, the inhibition of GBM proliferation by urolithins has rarely been explored.

Aryl hydrocarbon receptor (AhR), a ligand-activated transcription factor, responds to endogenous signals, xenobiotic chemicals, and the toxicity of dioxin-like chemicals [26,27]. AhR regulates downstream genes through transcriptional expression that modulates critical cellular activities, such as the induction of metabolizing enzymes and embryonic development [28,29]. AhR may be constitutively active in tumors and promote their development because it is overexpressed in various tumor types [30,31]. Moreover, AhR controls cancer cell survival and tumor-associated immune system functions [26]. Urolithins are considered an AhR antagonist [32]. Treatment with urolithin A reduces colitis through the AhR receptor [33]. Urolithin A also ameliorated experimental autoimmune encephalomyelitis [34] and carrageenan-induced paw edema in mice models [35] by targeting the AhR receptor.

This study elucidated the modulatory mechanism explaining the effects of urolithins on GBM motility and proliferation, and the effects of urolithins on the TME of GBM. We also assessed the inhibitory regulation of AhR in GBM progression.

## 2. Materials and Methods

### 2.1. Materials

Urolithin A, urolithin B, phorbol 12-myristate 13-acetate (PMA), 6,2′,4′-trimethoxyflavone (TMF), and primary antibodies against vimentin, α-tubulin, and GAPDH were obtained from Sigma-Aldrich (St. Louis, MO, USA). Primary antibodies against β-catenin, VCAM-1, and PD-L1 were obtained from Abcam (Cambridge, UK). Primary antibodies against *N*-cadherin, p-AKT, and p-STAT3 were acquired from Santa Cruz Biotechnology (Santa Cruz, CA, USA). The primary antibody against EGFR was obtained from Cell Signaling Technology (Danvers, MA, USA). BCEFC/AM was purchased from Invitrogen (Carlsbad, CA, USA).

### 2.2. Cell Culture

U251 human glioma cells were obtained from the JCRB NO. IFO50288, Japan. U87 human glioma, ALTS1C1 mouse glioma, and THP-1 human monocytes were obtained from the Bioresource Collection and Research Center (BCRC No. 60360, 60430 and 60582; Hsinchu, Taiwan). U251 and U87 cells were cultured in Minimum Essential Medium (MEM) supplemented with 10% FBS and 1% PS. THP-1 was cultured in RPMI-1640 medium with 10% FBS (fetal bovine serum), 1% PS, and 2-Mercaptoethanol. ALTS1C1 was cultured in Dulbecco’s MEM. All cell lines were cultured at 37 °C in a humidified incubator containing 5% CO_2_ and 95% air.

### 2.3. Migration Assay

U251 and ALTS1C1 GBM cells were pretreated with urolithin A or urolithin B for 24 h. Then, 1.5 × 10^4^ cells were seeded in the Culture-Insert (Ibidi, München, DE, Germany). The insert was removed after 4 h of culture. Cell migration was detected and photographed using a light microscope and a digital camera at 0 and 24 h after the removal of the insert. Transmigration was performed according to our previous study [36]. Briefly, 5 × 10^4^ cells were plated in the Costar transwell insert (Costar, NY, USA). The migrated cells were stained with crystal violet and photographed using a microscope with digital camera.

### 2.4. Western Blotting

To prepare total protein lysates, radioimmunoprecipitation assay (RIPA) cell lysis buffer, containing protease and phosphatase inhibitor cocktails, was added to the cells. Then, cells were collected using a cell scraper and kept on ice for 10 min. The samples were centrifuged for 20 min at 12,000 rpm and 4 °C. Supernatants were collected and stored at −20 °C. Protein samples (30 μg) were denatured in Laemmli buffer at 95 °C for 5 min, separated using SDS-page electrophoresis, and transferred to polyvinylidene difluoride (PVDF) membranes (Millipore, Bedford, MA, USA). The membranes were blocked with 5% non-fat milk in tris-buffered saline buffer with tween 20. Membranes were washed three times and incubated with primary antibodies at 4 °C overnight followed by secondary antibodies at room temperature for another 1 h. The antibodies were removed and washed several times. The blots were visualized by ECL and Kodak X-OMAT LS film (Eastman Kodak, Rochester, NY, USA). The quantitative results were obtained using ImageJ software 1.53t (National Institutes of Health, Bethesda, MD, USA).

### 2.5. Colony Formation

A total of 1 × 10^3^ GBM cells were seeded on the 3.5 cm dish and treated with urolithin A or urolithin B for 14 days. Medium containing urolithins was renewed every three days. Cells were stained with 0.1% crystal violet for 1 h. Then, cell colony formation was detected and photographed using a digital camera.

### 2.6. Cell Viability Assay

Cell viability was determined by 3-(4,5-dimethylthiazol-2-yl)-2,5-diphenyltetrazolium bromide (MTT) assay. The procedure of MTT assay was performed according to our previous report [37].

### 2.7. Monocyte Binding Assay

The procedure of monocyte binding assay was performed according to our previous report [38]. Briefly, GBM cells were treated with urolithin A or urolithin B for 24 h. THP-1 cells were stained with 200 ng/mL BCEFC/AM for 1 h at 37 °C. The excess of BCEFC/AM was washed twice using a fresh culture medium. The monolayer of GBM cells were added to the BCEFC/AM-labeled THP-1 (3 × 10^5^ cells) and incubated at 37 °C for 30 min. Then, the medium and non-binding monocyte cells were removed and washed gently with fresh culture medium twice. The adherent THP-1 cells were captured by a fluorescence microscope and analyzed by the Image J software.

### 2.8. Human Macrophage Conditioned Medium (HMCM) Collection

THP-1 monocytes (5 × 10^5^) were seeded on 3.5 cm dishes and treated with 100 nM PMA for 3 days. The spent medium was replaced with fresh medium. Cells were allowed to rest for 1 day. After 3 days of culture, the conditioned medium was assembled and stored at −80 °C for future treating of GBM cells.

### 2.9. Cell Transfection

GBM cells were seeded on 6-well plate (3 × 10^5^ cells/well). The smart pool siRNA against non-targeting control or AhR were premixed with serum-free medium and DharmaFECT transfection reagents (Dharmacon, Lafayette, CO, USA) and then applied to the cells.

### 2.10. ELISA Assay

A human soluble VCAM-1 DuoSet ELISA kit was purchased from R&D Systems (Minneapolis, MN, USA) and used for the quantification of soluble VCAM-1 protein secreted to the supernatant of GBM culture. The assay was performed according to the manufacturer’s instructions. The results were determined using the EPOACH2 microplate reader (Bio-Tek, Winooski, VT, USA) at OD 450 nm.

### 2.11. Tumor Xenograft

All animal studies were performed in accordance with the Institutional Animal Care and Use Committee (IACUC) of China Medical University. Male C57BL/6 mice (10-week-old) were obtained from the National Laboratory Animal Center (Taipei, Taiwan), and kept with water and food consumption ad libitum under standard laboratory conditions. A total of 1 × 10^7^ mouse ALTS1C1 GBM cells were dissociated in 100 μL PBS, and were subcutaneously inoculated into the rear flank. Urolithin A (40 mg/kg) and vehicle were orally administered once per day. Tumor volume was measured 7 days after tumor transplantation.

### 2.12. Statistics

Results are displayed as the mean ± S.E.M. All the data were performed with at least three biologically independent replicates. The significant difference between two samples was assessed using a Student’s *t*-test. * *p*-values < 0.05 were supposed significant and indicated in the figure legends. Statistical analysis was implemented by using SigmaPlot software (version 10.0, Systat Software Inc., San Jose, CA, USA).

## 3. Result

### 3.1. Urolithins Inhibit Migration Ability and Cell Proliferation in GBM

**Figure 1:**
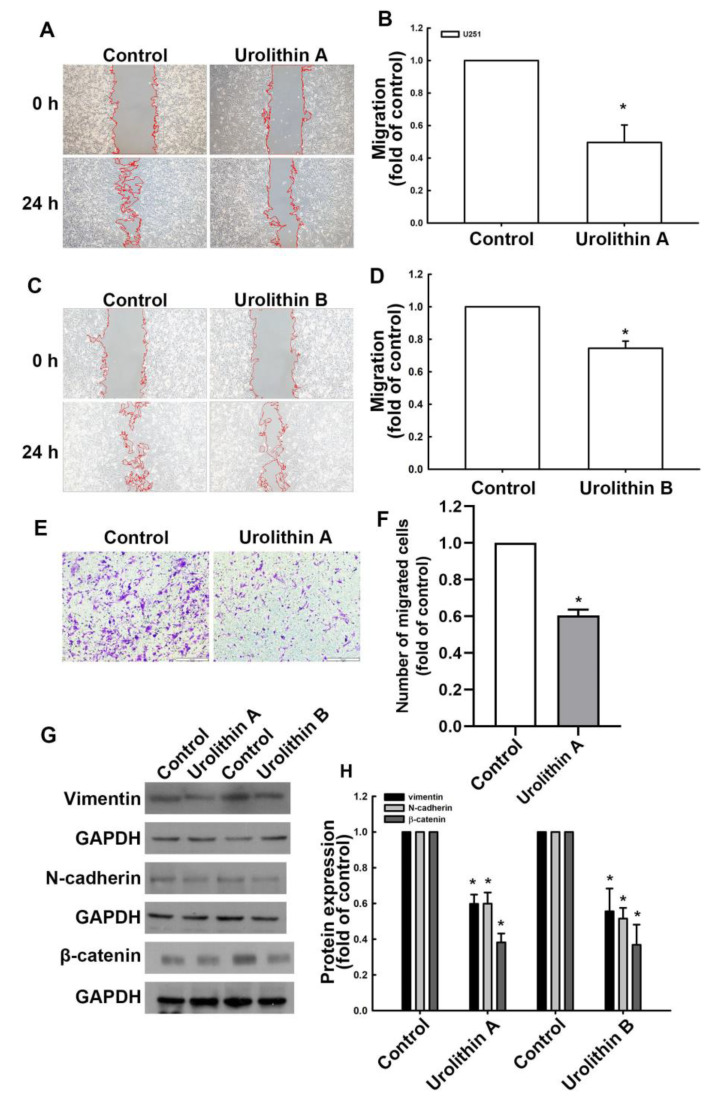
Inhibition of GBM migration and epithelial–mesenchymal transition by urolithin A and urolithin B. U251 cells were incubated with urolithins for 24 h. Migration ability was determined by a wound healing assay and visualized using a digital camera following treatment with urolithin A (**A**,**B**) and B (**C**,**D**). Transmigration ability was determined using a transwell assay and visualized using a microscope following treatment with urolithin A (**E**,**F**). Vimentin, *N*-cadherin, and β-catenin protein expression levels were determined by Western blotting (**G**,**H**). Each bar represents the mean ± standard error of the mean (*n* = 3). * *p* < 0.05 compared with the control group.

**Figure 2:**
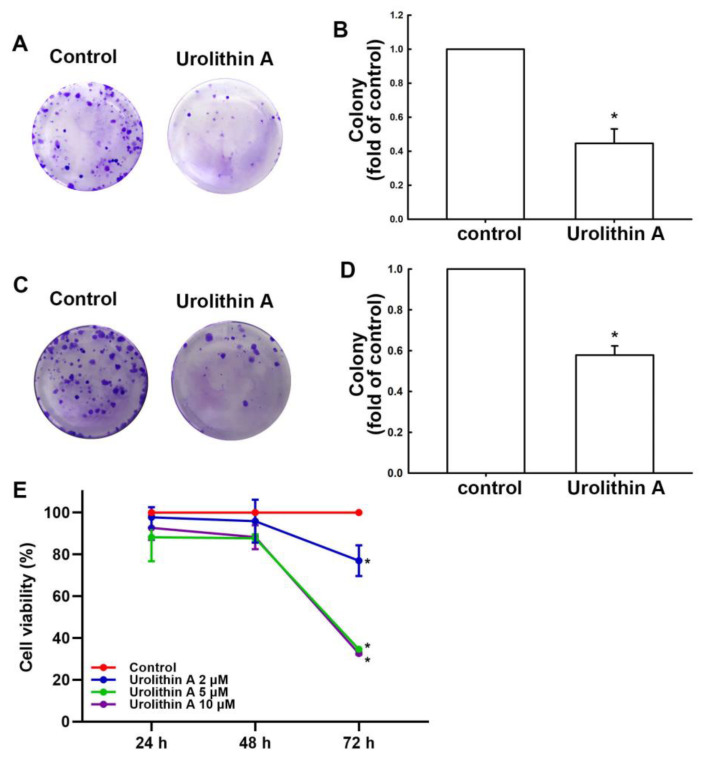
Urolithin A and urolithin B inhibit GBM cell growth. U251 human (**A**) and ALTS1C1 mouse (**C**) GBM cells were administrated with urolithin A for 14 days. Medium containing urolithin A was replaced every 3 days. Cells were incubated with 0.1% crystal violet for 1 h. Colony formation was captured using a microscope. Quantitative results shown in (**B**,**D**). (**E**) U251 human GBM cells were treated with various concentrations of urolithin A (2, 5 and 10 μM) for 24, 48, and 72 h. The cell viability was detected by MTT assay. Each bar represents the mean ± standard error of the mean (*n* = 3). * *p* < 0.05 compared with the control group.

During the development of GBM, cancer cells can migrate to distant areas and exhibit metastatic activity caused by the epithelial–mesenchymal transition (EMT) proteins [39]. In this study, we demonstrated that human GBM cells exhibited increased motility at 24 h post-culture compared with at 0 h in a wound healing assay (Figure 1A,C). Moreover, treatment with urolithin A (Figure 1A,B) and urolithin B (Figure 1C,D) for 24 h markedly reduced the migration ability of GBM cells. Furthermore, the transmigration ability of GBM cells was suppressed by urolithin A treatment as well (Figure 1E,F and Supplementary Figure S1A,B). We examined the expression of EMT-associated proteins as a contributors to cell motility in GBM cells following urolithin treatment. As shown in Figure 1G,H, the protein expression of vimentin, *N*-cadherin, and β-catenin was substantially reduced by treatment with urolithin A (Figure 1G,H and Supplementary Figure S1C,D). Treatment with urolithin B also attenuated vimentin, *N*-cadherin, and β-catenin protein expression (Figure 1G,H and Supplementary Figure S1C,D). Effects of urolithin A on GBM proliferative activity was further determined. In Figure 2A,B, the colony formation in human GBM cells without any treatment was greater than in GBM cells treated with urolithin A. Furthermore, the colony formation was inhibited by urolithin A in mouse GBM cells (Figure 2C,D). In addition, urolithins also suppressed the GBM cells proliferation after 72 h treatment (Figure 2E). This indicates that urolithins effectively reduce cell motility and viability in GBM cells.

### 3.2. Urolithins Reduce Expression of VCAM-1 and PD-L1 in GBM Cells

**Figure 3:**
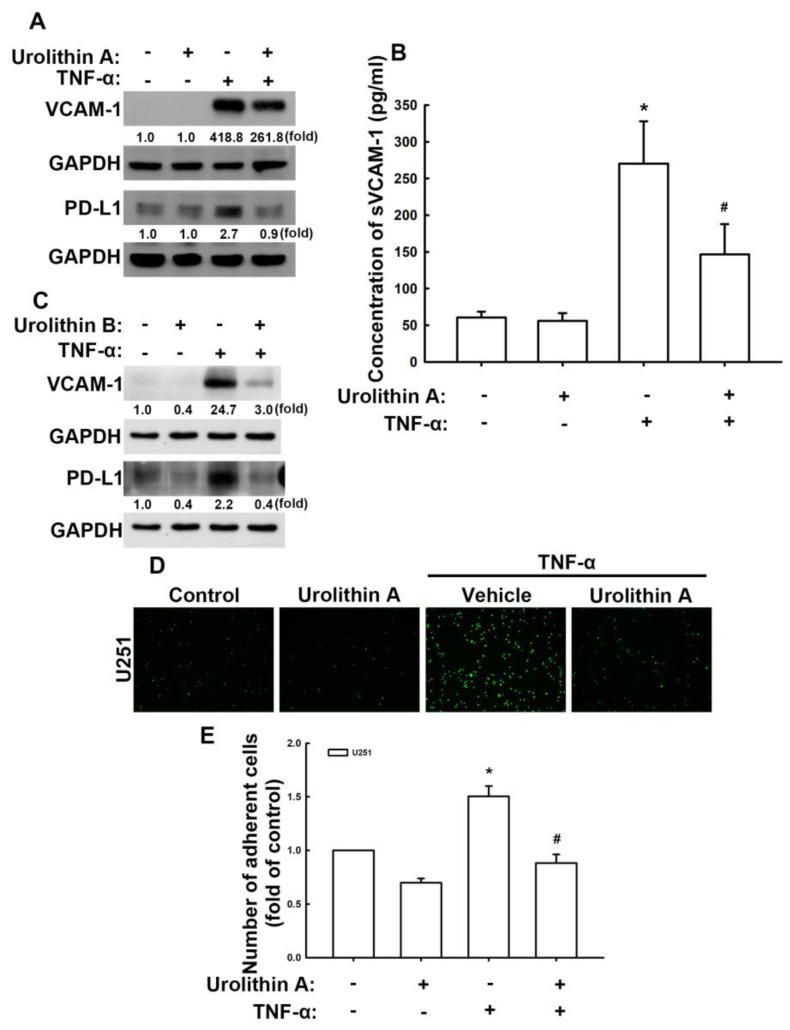
Urolithins inhibit tumor necrosis factor (TNF)-α-induced expression of vascular cell adhesion molecule (VCAM)-1 and programmed death ligand (PD-L1) and reduce monocyte adhesion in GBM. U251 human GBM cells were treated with urolithins for 30 min and stimulated with TNF-α for another 24 h. Expression of VCAM-1 and PD-L1 were determined using Western blotting following treatment with urolithin A (**A**) and B (**C**). (**B**) After 24 h of TNF-α stimulation, fresh serum-free medium was replaced and cultured for another 24 h. Supernatants were collected. sVCAM-1 expression was determined by using ELISA assay. (**D**) U251 GBM cells were pretreated with urolithin A for 30 min and stimulated with TNF-α for another 24 h. Then BCECF-AM-labeled THP-1 cells were added to GBM cells for 30 min. Quantitative results shown in (**E**). Each bar represents the mean ± standard error of the mean (*n* = 3). * *p* < 0.05 compared with the control group. ^#^ *p* < 0.05 compared with the TNF-α group.

We previously have reported that proinflammatory cytokines, which are abundantly produced in the TME, regulated the expression of VCAM-1, thus promoting the invasion of monocytes or macrophages to GBM cells and worsening GBM outcomes [38,40]. Here, we used tumor necrosis factor (TNF)-α to promote the expression of VCAM-1 in GBM cells to evaluate the anticancer effects of urolithins. TNF-α stimulates the expression of VCAM-1 protein in U251 human GBM cells (Figure 3A,C) and U87 GBM cells (Supplementary Figure S2A). Treatment with urolithin A (Figure 3A) and B (Figure 3C) sharply reduced TNF-α-enhanced VCAM-1 protein expression in U251 and U87 (Supplementary Figure S2A) GBM cells. Furthermore, TNF-α markedly enhanced the expression of PD-L1 proteins in U251 (Figure 3A,C) and U87 (Supplementary Figure S2A) GBM cells, which was reversed following treatment with urolithin A (Figure 3A) or urolithin B (Figure 3C). We also detected soluble VCAM-1 in U251 human GBM. Stimulation of TNF-α expression significantly enhanced soluble VCAM-1 expression (Figure 3B). Urolithin A administration also reduced the enhancement of soluble VCAM-1 expression (Figure 3B). Subsequently, the ability of monocytes to bind to GBM was determined using a monocyte-binding assay. Stimulation of TNF-α enhanced monocyte adhesion on GBM cells (Figure 3D,E). Treatment with urolithin A significantly reduced TNF-α-induced monocyte adhesion to GBM cells (Figure 3D,E). These results indicate that urolithins effectively attenuate VCAM-1 and PD-L1 expression, thereby reducing monocyte adhesion to GBM cells.

### 3.3. Involvement of Adhesion Molecules in AhR Regulation of GBM Progression

**Figure 4:**
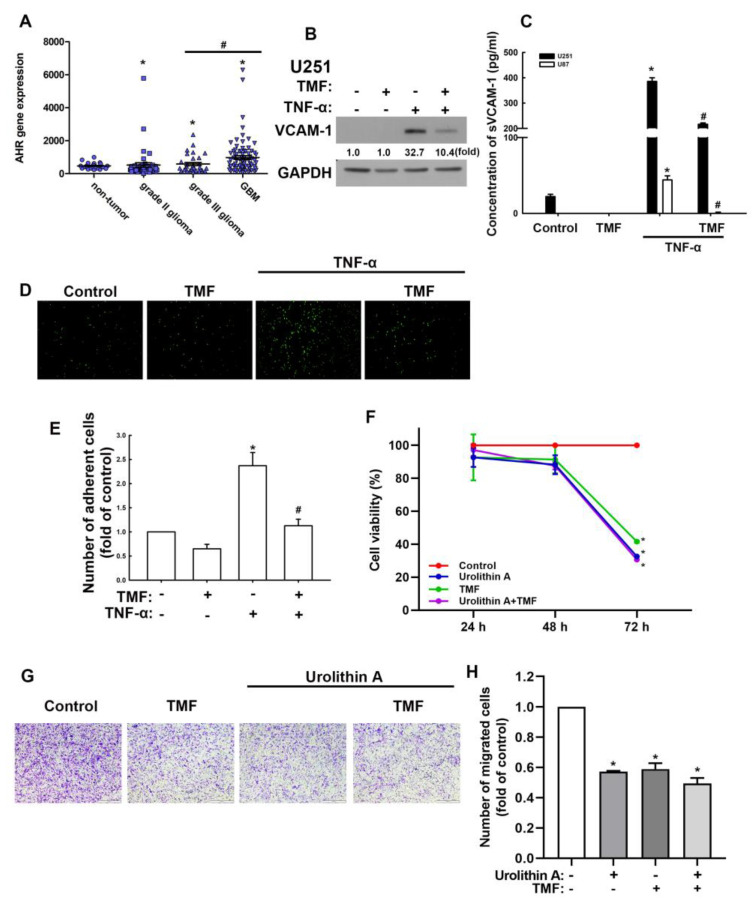
Aryl hydrocarbon receptor (AhR) expression in GBM is correlated with pathologic grades of human glioma and involves adhesion molecule expression. (**A**) AhR mRNA levels of patients’ specimens from human glioma microarray data set GSE4290. (**B**) U251 human GBM was treated with AhR antagonist 6,2′,4′-trimethoxyflavone (TMF) and stimulated with tumor necrosis factor (TNF)-α for another 24 h. Vascular cell adhesion molecule (VCAM)-1 expression was evaluated by Western blotting. (**C**) U251 and U87 GBM cells were treated with TMF for 30 min and stimulated with TNF-α for another 24 h. The cultured medium was replaced with fresh serum-free medium. Supernatants were collected after 24 h. Soluble VCAM-1 expression was determined using enzyme-linked immunosorbent assay. (**D**) U251 GBM cells were pretreated with TMF for 30 min and stimulated with TNF-α for another 24 h. BCECF-AM-labeled THP-1 cells were added to GBM cells and cultured for 30 min. THP-1 adhesion to GBM cells was observed under a fluorescence microscope. Quantitative results are shown in (**E**). (**F**) U251 human GBM cells were treated with urolithin A (10 μM), TMF (10 μM), or cotreatment for 24, 48, and 72 h. Cell viability was performed using MTT assay. (**G**,**H**) U251 human GBM cells were treated with urolithins and TMF for 24 h; cell migration was detected by using transwell assay. Each bar represents the mean ± standard error of the mean. * *p* < 0.05 compared with the control group. ^#^ *p* < 0.05 compared with the TNF-α group.

**Figure 5:**
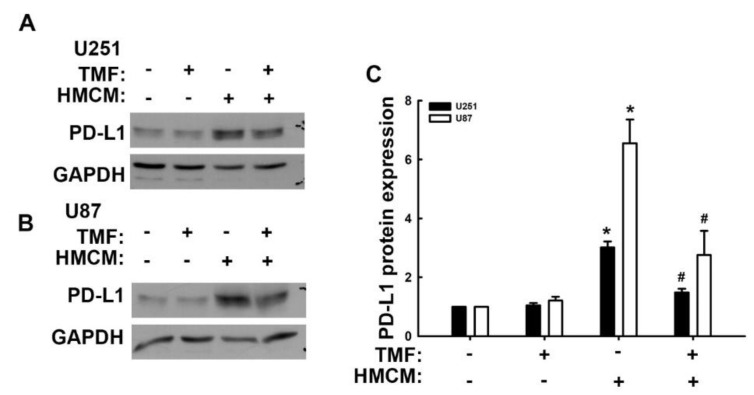
Inhibition of aryl hydrocarbon receptor using 6,2′,4′-trimethoxyflavone (TMF) represses human macrophage condition medium (HMCM)-induced programmed death ligand (PD-L1) expression in GBM cells. U251 (**A**) and U87 (**B**) human GBM were treated with TMF for 30 min and stimulated with HMCM for another 24 h. PD-L1 expression was determined using Western blotting. Quantitative results are shown in (**C**). Each bar represents the mean ± standard error of the mean (*n* = 3). * *p* < 0.05 compared with the control group. ^#^ *p* < 0.05 compared with the HMCM group.

First, we assessed whether the modulatory effects of urolithins in GBM involve AhR expression. First, we analyzed AhR gene expression using the human glioma microarray data set GSE4290. AhR expression in patients with all grades of glioma (grades II and III and GBM) was higher than that of patients without tumors and the level of expression correlated with the clinical grades (Figure 4A), indicating the regulatory role of AhR in GBM. Subsequently, we used an AhR-selective pharmacological antagonist, 6,2′,4′-trimethoxyflavone (TMF), to study the effects of AhR on GBM progression. As shown in Figure 4B, treatment with TMF markedly reduced the enhancement of VCAM-1 protein expression induced by TNF-α. TMF treatment also reduced TNF-α-induced soluble VCAM-1 expression in both human U251 and U87 GBM (Figure 4C). The administration of TMF alone did not affect monocytes binding to GBM cells (Figure 4D,E). TNF-α-enhanced monocyte GBM adhesion was reduced following TMF treatment (Figure 4D,E). The cell viability of GBM was not changed with urolithin A, TMF, or cotreatment until 48 h (Figure 4F). But the cell viability was decreased when GBM cells were treated with urolithin A, TMF, or cotreatment at 72 h (Figure 4F). In addition, cotreatment of urolithin A and TMF did not exacerbate the GBM death (Figure 4F). Moreover, treatment of urolithin A or TMF also reduced the migration of GBM cells, but the treatment of urolithin A and TMF did not reduce migration more than the treatment alone group (Figure 4G,H). Additionally, we used small interfering RNA (siRNA) against AhR to confirm the anti-GBM effects of urolithins. The TNF-α-induced VCAM-1 and PD-L1 expression were reduced when transfected with AhR siRNA (Supplementary Figure S2B). In addition, the GBM migration were also inhibited by transfection with AhR siRNA or urolithin A treatment (Supplementary Figure S2C,D). However, the inhibitory effects of AhR siRNA with GBM migration did not affect urolithin A cotreatment (Supplementary Figure S2C,D). Furthermore, we previously reported that the secreted factors from macrophages may modulate the TME of GBM. We discovered that PD-L1 is expressed in GBM through a contact-free mechanism [41]. In the present study, we assemble the human macrophage condition medium (HMCM) and used it to treat human GBM and assess PD-L1 expression. Treatment with HMCM enhanced PD-L1 expression in both human U251 (Figure 5A,C) and U87 (Figure 5B,C) GBM cells. Treatment with TMF decreased HMCM-promoted PD-L1 expression in U251 GBM cells (Figure 5A,C). A similar result was observed in U87 GBM cells (Figure 5B,C). In addition, the administration of TMF alone did not affect expression of PD-L1 in GBM (Figure 5A–C). Our results suggest that AhR is a critical modulator in GBM progression, and urolithins may act on AhR to regulate the TME of GBM.

### 3.4. Akt and Epidermal Growth Factor Receptor Signaling Pathways Are Involved in Urolithin A Inhibition of GBM Progression

**Figure 6:**
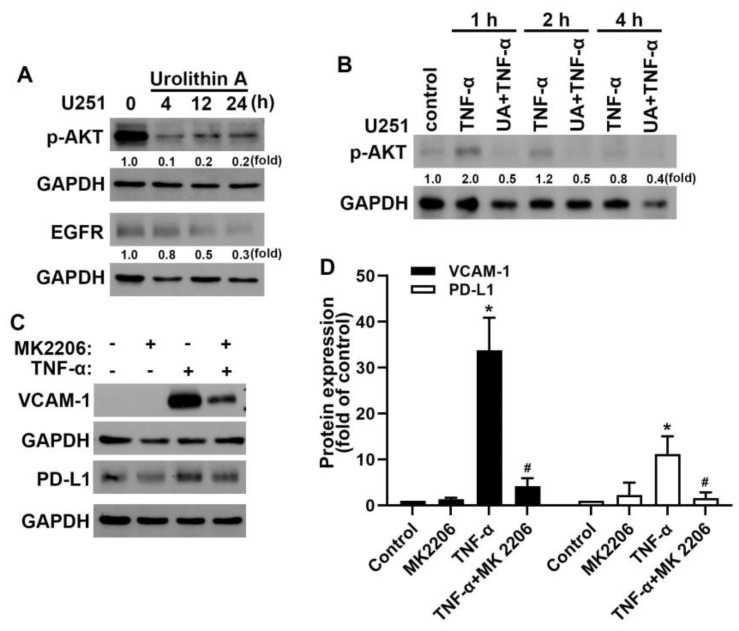
Urolithin A inhibits Akt/epidermal growth factor receptor (EGFR) activation in GBM cells. (**A**) U251 human GBM cells were treated with urolithin A for indicated time periods (4, 8, 12, or 24 h). Expression of p-Akt and EGFR proteins was determined using Western blotting. (**B**) U251 cells were treated with tumor necrosis factor (TNF)-α for 1, 2, or 4 h. Thirty minutes before the end of each time point, urolithin A was added to the cells. Levels of p-Akt and p-EGFR proteins were quantified using Western blotting. (**C**) U251 GBM cells were treated with Akt inhibitor MK2206 (1 μM) for 30 min and TNF-α for another 24 h. Vascular cell adhesion molecule 1 and programmed death ligand protein expression were evaluated by Western blotting. Quantitative results are shown in (**D**). Each bar represents the mean ± standard error of the mean (*n* = 3). * *p* < 0.05 compared with the control group. ^#^ *p* < 0.05 compared with the TNF-α group.

Our previous study demonstrated that Akt [36,38] and epidermal growth factor receptor (EGFR) [36,40] pathways are critical in TME and GBM progression. We further examined the signaling pathways involved in the inhibition of GBM by urolithins. In this study, we demonstrated that treatment of GBM cells with urolithin A caused a reduction in phosphorylated Akt and EGFR protein expression in a time-dependent manner (Figure 6A). Urolithin A also reduced TNF-α-induced Akt phosphorylation (Figure 6B). We further determined whether urolithin A regulated VCAM-1 and PD-L1 through the Akt signaling pathway. Treatment with Akt inhibitor MK2206 reduced the expression of TNF-α-induced VCAM-1 and PD-L1 (Figure 6C,D). Taken together, our results suggest that urolithins inhibit GBM progression by modulating Akt and EGFR signaling pathways.

### 3.5. Treatment with Urolithins Suppress the GBM Growth in Xenograft Mouse Model

**Figure 7:**
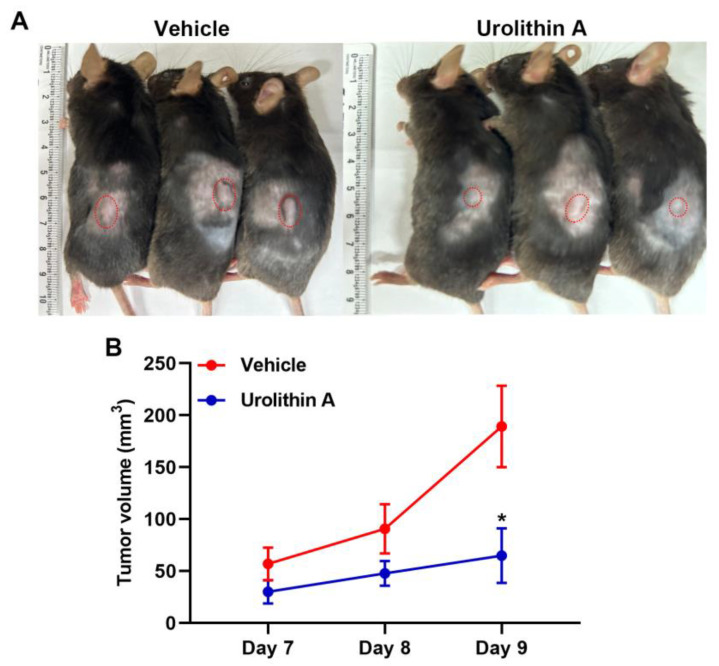
Urolithins inhibit the GMB growth in xenograft mouse model. (**A**) Mice ALTS1C1 GBM cells (1 × 10^7^ cells) were injected subcutaneously into the flank of each mouse. Urolithin A (40 mg/kg) and vehicle were intraperitoneally injected once per day. (**B**) Tumor volumes were measured and calculated every day after tumor injection. Quantitative data are presented as mean ± SEM. * *p* < 0.05 compared with the vehicle control (Student’s *t*-test).

To verify the influences of urolithins on GBM progression in vivo, we implanted mouse ALTS1C1 GBM cells into mice and treated with urolithins. We observed that treatment with urolithin A decreased the tumor growth compared to the vehicle group (Figure 7A). The tumor volume in the vehicle group was markedly increased but only mild increased with urolithin A treatment (Figure 7B). These findings suggest that urolithins treatment reduces tumor growth and exerts anti-GBM effects.

## 4. Discussion

In the TME, the proinflammatory cytokine TNF-α is mainly produced by macrophages and other immune cells, including dendritic cells, B cells, natural killer cells, and T-cells [42]. TNF-α is closely related to tumor metastasis. It stimulates the expression of several angiogenic factors such as interleukin-8, basic fibroblast growth factor, and vascular endothelial growth factor that contribute to the progression and expansion of tumors [43]. In addition, TNF-α was reported to induce EMT and promote the metastasis and invasion of colorectal cancer [44]. Accumulating evidence suggests that TNF-α is involved in the expression of VCAM-1 and GBM metastasis [45]. We previously found that TNF-α promoted the adhesion molecule expression and supported monocyte binding to GBM [40]. Urolithin A was shown to ameliorate TNF-α-stimulated endothelial cell migration and reduce monocyte adhesion to endothelial cells [46]. Moreover, GBM is considered to have immunosuppressive effects because of the inhibition of immune cell functions caused by endogenous immunosuppressive checkpoints produced such as PD-L1 [47,48]. Targeting the PD-1/PD-L1 signaling pathway enables blocking of this inhibitory signal and enables T-cells to attack tumor cells. The beneficial effects of urolithin A in cancer therapy are evident; it strongly enhances antitumor T-cell (CD8^+^) immunity in colorectal cancer, making it an effective immune checkpoint blockade treatment option for cancer [49]. Our results support previous studies demonstrating that urolithin treatment inhibits the TNF-α-enhanced VCAM-1 expression and prevents monocytes from adhering to GBM. The present study also supports these findings by demonstrating that urolithins reduce the induction of PD-L1 expression, thus inhibiting GBM progression.

Extensive tumor proliferation and infiltrative expansion to neighboring tissues are among the major clinical hallmarks of GBM [50]. EMT has emerged as a contributor to the invasiveness of cancer cells. EMT transformation results in the alteration of epithelial and mesenchymal markers, particularly E-cadherin, *N*-cadherin, vimentin, and fibronectin, which promote the ability of tumor cells to migrate and spread to nearby or distant areas [51]. Several effective anticancer drugs target the relative molecules of cancer cell growth and motility [52,53,54]. The use of phytochemicals as an anticancer agent has gained increased attention because of their accessibility, patient safety, and efficacy [55]. In particular, urolithin B was reported to inhibit the progression of hepatocellular carcinoma by inactivating the β-catenin signaling pathway [56]. In this study, we demonstrated that treatment with urolithin A or B markedly suppressed the cell motility factors such as colony formation and migration ability of GBM cells. Urolithin A and B treatment in GBM cells may prevent the expression of mesenchymal molecules.

AhR regulates the expression of cytochromes P450, which are the major enzymes responsible for tumor formation, metastasis, and prevention [26,27,57]. AhR has been a target of cancer therapy drug development [58]. AhR inhibition using pharmacological agents and gene silencing reduced clonogenic survival and invasiveness of glioma cells [27]. A study also showed that AhR modulated GBM migration ability [59]. Endogenous AhR activity is positively correlated with cell migration in human GBM cells [59]. Generally, humans and animals are exposed to both synthetic and natural AhR ligands mostly through their diet. Several natural dietary compounds were reported to activate or inhibit the AhR signaling pathways [60]. Urolithin A is an antagonist of AhR [32]. Kujawska et al. demonstrated that urolithin A attenuates the chemically induced stimulation of CYP1A1 mRNA, which is involved in antagonizing AhR [61]. In this study, we demonstrated that treatment with the AhR antagonist TMF ameliorated TNF-α-enhanced VCAM-1 protein levels, monocyte binding ability, and HMCM-stimulated PD-L1 protein expression on human GBM cells. Analysis of the human glioma microarray dataset GSE4290 demonstrated a positive correlation between AhR expression and pathological glioma grade. In addition, the GBM migration were also inhibited by knockdown of AhR in GBM. Our results suggest a role of AhR in the development of GBM and provide a regulation of urolithins that may inhibit GBM progression by modulating the AhR signaling pathway.

**Figure 8:**
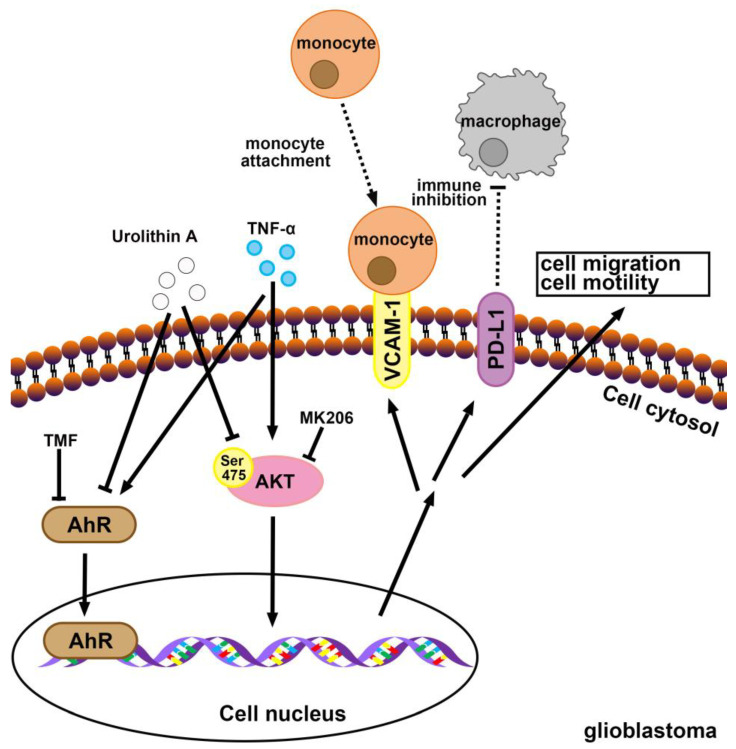
Schematic diagrams of urolithin A signaling pathways that inhibit GBM progression. Tumor necrosis factor (TNF)-α promotes the vascular cell adhesion molecule (VCAM)-1 levels and promotes monocyte binding to GBM. Furthermore, TNF-α promotes the programmed death ligand (PD-L1) expression. Urolithin A and aryl hydrocarbon receptor antagonist 6,2′,4′-trimethoxyflavone suppress TNF-α-enhanced expression of VCAM-1 and PD-L1. Urolithin A modulates the Akt signaling pathway to prevent tumor migration and growth.

We have recently reported that Akt signaling is critical for GBM progression [38]. Wang et al. have reported that expression of PD-L1 in human colon and prostate cancers was stimulated by TNF-α through the activation of Akt signaling [62]. It has also been reported that urolithin A inhibits the progression of pancreatic cancer [24] and glioma [63] by blocking the phosphorylation of Akt. EGFR alteration occurs in more than half of patients with GBMs [64,65] and is also correlated with GBM progression [66,67]. High EGFR expression in patients with GBM is associated with poor outcomes [68,69,70]. In clinical studies, treatment with gefitinib and erlotinib, EGFR tyrosine kinase inhibitors, produced promising results in patients with GBM [71]. Zheng et al. [72] and our team [40] have also reported that the activation of EGFR modulates the expression of VCAM-1 in GBM. We previously demonstrated that TNF-α-induced EGFR activation is important for modulating the TME of GBM [40]. Recently, a network pharmacology analysis suggested that urolithins may act on the EGFR and Akt signaling for anti-abnormal uterine bleeding effect [73]. The present study corroborated that urolithins reduce the TME and inhibit tumor progression in GBM cells by regulating Akt and EGFR signaling pathways (Figure 8).

## 5. Conclusions

This study investigated the inhibitory role of urolithin, the bioactive gut metabolite from natural polyphenols, against GBM. We demonstrated that urolithins suppress GBM colony formation and cell migration. Urolithins further reduced TNF-α-stimulated VCAM-1 and PD-L1 expression, thereby lessening monocyte binding to GBM. We also demonstrated that urolithin A regulates the inhibition of GBM progression through Akt activation and EGFR expression. In addition, the TNF-α-induced expression of VCAM and PD-L1 was reduced when AhR signaling was inhibited. These results indicate that TNF-α may induce the expression of VCAM and PD-L1 through the regulation of AhR. Finally, administration of urolithin A inhibited the tumor growth in vivo. Consequently, our findings indicate that urolithins could be used as a promising strategy for the effective management of GBM.

## Data Availability

The data that support the findings are not publicly available. Data are available from the authors upon reasonable request.

## Supplementary Materials

The following supporting information can be downloaded at: https://www.mdpi.com/article/10.3390/nu15234854/s1, Figure S1: Inhibitory effects of GBM migration and epithelial–mesenchymal transition by urolithins in ALTS1C1 GBM cells; Figure S2: Knockdown of aryl hydrocarbon receptor downregulates the expression of VCAM-1 and PD-L1.
