# Supplementary material for: Inhibitory Effects of Urolithins, Bioactive Gut Metabolites from Natural Polyphenols, against Glioblastoma Progression
Source: Nutrients. 2023 Nov 21;15(23):4854. doi: 10.3390/nu15234854 (PMC10708538; doi:10.3390/nu15234854)
Supplement: Supplementary file 1 [file nutrients-15-04854-s001.zip › nutrients-2685498-supplementary.pdf]

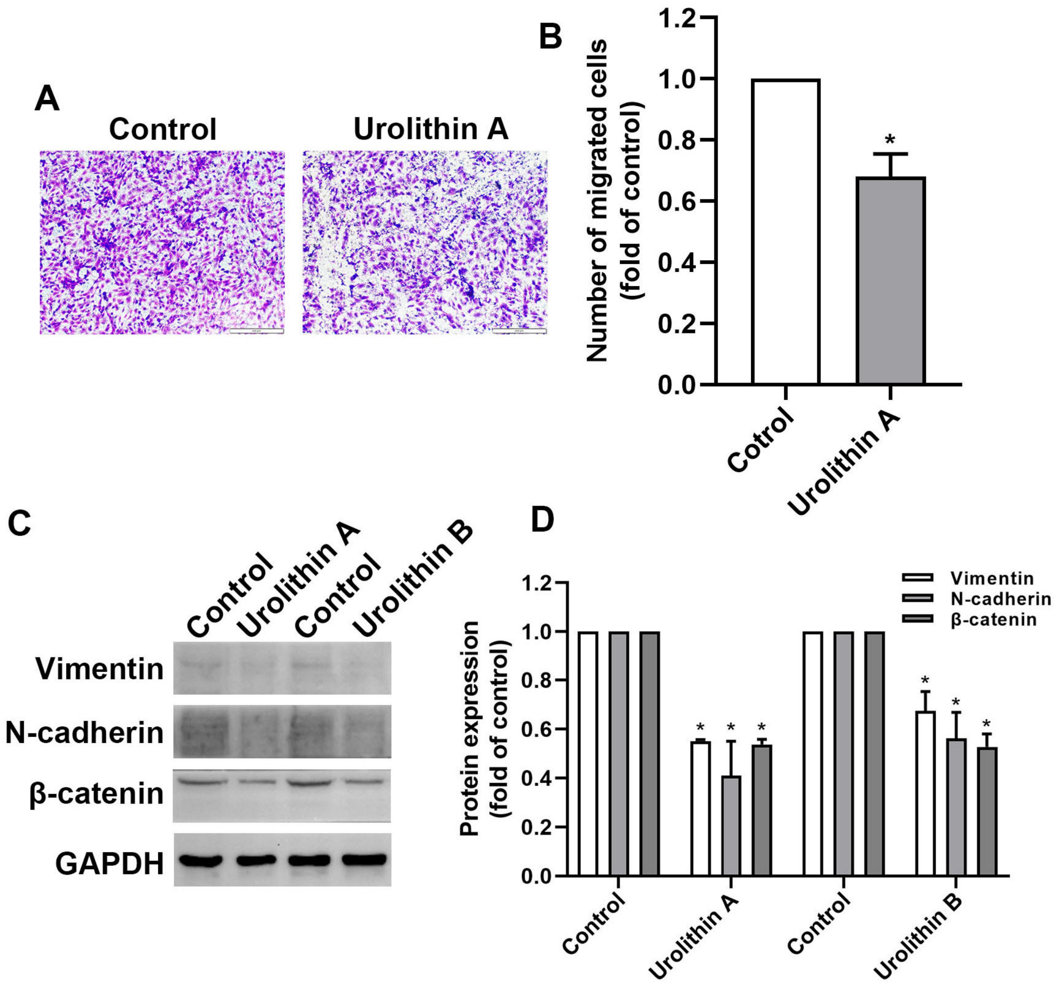

Figure S1. Inhibitory effects of GBM migration and epithelial–mesenchymal transition by urolithins in ALTS1C1 GBM cells. (A, B) Cells were treated with urolithin A for 24 h. Migration ability was determined using a transwell assay and visualized using a microscope. (C, D) Vimentin, N-cadherin, and β-catenin protein expression levels were determined by Western blotting. Each bar represents the mean ± standard error of the mean (n = 3) *$p < 0.05$ compared with the control group.

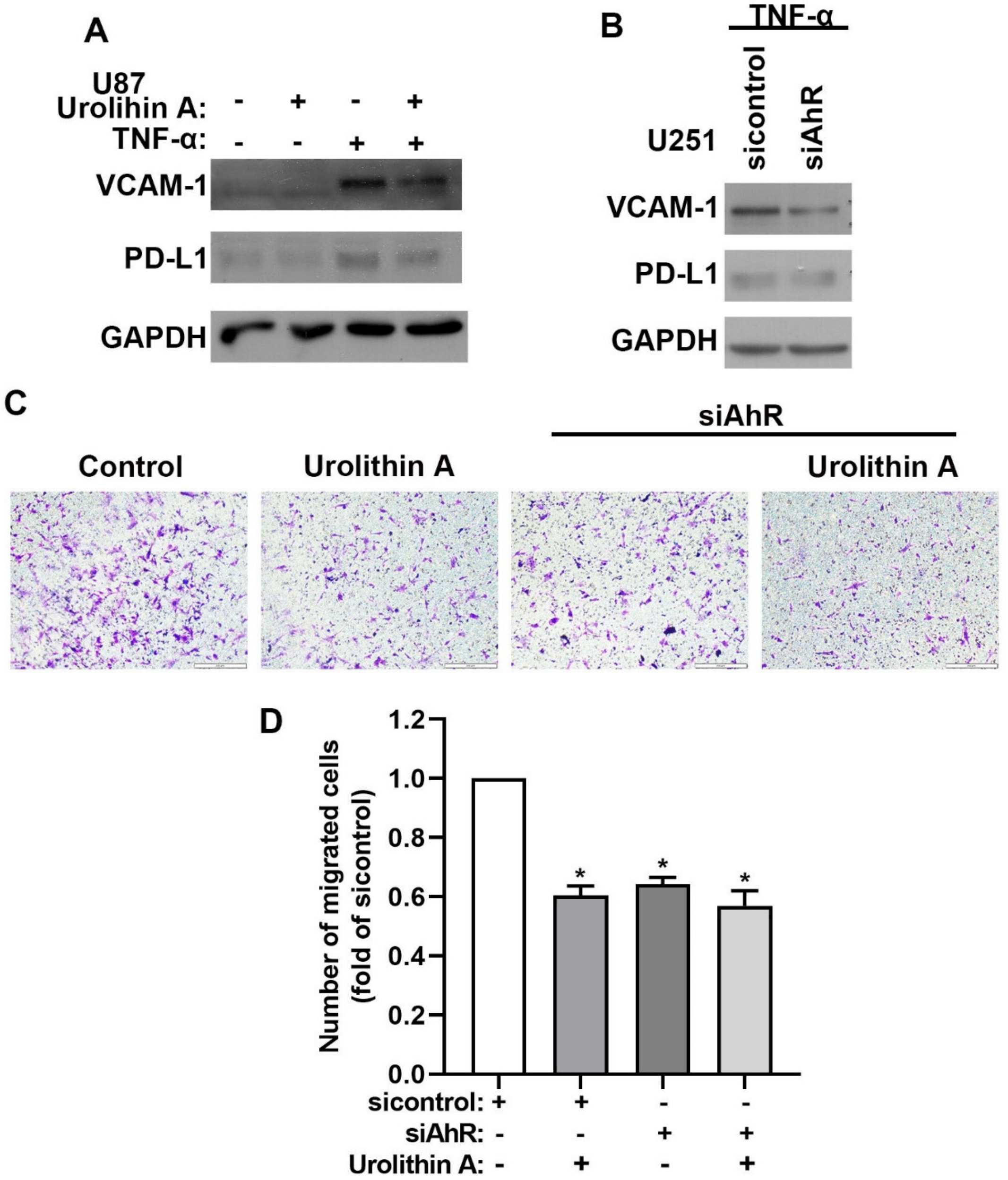

Figure S2. Knockdown of aryl hydrocarbon receptor downregulates the expression of VCAM-1 and PD-L1. (A) U87 human GBM cells were treated with urolithin A for 30 min and stimulated with TNF-α for another 24 h. (B) U251 human GBM cells were transfected with siRNA against AhR or control for 24 h and then stimulated with TNF-α for another 24 h. VCAM-1 and PD-L1 protein expression levels were determined using Western blotting. (C) U251 human GBM cells were transfected with AhR or control siRNA for 24 h and stimulated with urolithin A for another 24 h. Cell migration was detected by using transwell assay. Quantitative results are shown in (D). Each bar represents the mean ± standard error of the mean (n = 3) $*p < 0.05$ compared with the sicontrol group.
